# Field evaluation of a 0.005% fipronil bait, orally administered to *Rhombomys opimus*, for control of fleas (Siphonaptera: Pulicidae) and phlebotomine sand flies (Diptera: Psychodidae) in the Central Asian Republic of Kazakhstan

**DOI:** 10.1371/journal.pntd.0006630

**Published:** 2018-07-25

**Authors:** David M. Poché, Zaria Torres-Poché, Aidyn Yeszhanov, Richard M. Poché, Alexander Belyaev, Vit Dvořák, Zaure Sayakova, Larisa Polyakova, Batirbek Aimakhanov

**Affiliations:** 1 Genesis Laboratories, Inc. Wellington, Colorado, United States of America; 2 M. Aikimbaev’s Kazakh Science Centre for Quarantine of Zoonotic Diseases. Almaty, Kazakhstan; 3 Department of Parasitology, Charles University, Prague, Czech Republic; Saudi Ministry of Health, SAUDI ARABIA

## Abstract

Plague (*Yersinia pestis*) and zoonotic cutaneous leishmaniasis (*Leishmania major*) are two rodent-associated diseases which are vectored by fleas and phlebotomine sand flies, respectively. In Central Asia, the great gerbil (*Rhombomys opimus*) serves as the primary reservoir for both diseases in most natural foci. The systemic insecticide fipronil has been previously shown to be highly effective in controlling fleas and sand flies. However, the impact of a fipronil-based rodent bait, on flea and sand fly abundance, has never been reported in Central Asia. A field trial was conducted in southeastern Kazakhstan to evaluate the efficacy of a 0.005% fipronil bait, applied to gerbil burrows for oral uptake, in reducing *Xenopsylla* spp. flea and *Phlebotomus* spp. sand fly abundance. All active gerbil burrows within the treated area were presented with ~120 g of 0.005% fipronil grain bait twice during late spring/early summer (June 16, June 21). In total, 120 occupied and 14 visited gerbil colonies were surveyed and treated, and the resulting application rate was minimal (~0.006 mg fipronil/m^2^). The bait resulted in 100% reduction in *Xenopsylla* spp. flea abundance at 80-days post-treatment. Gravid sand flies were reduced ~72% and 100% during treatment and at week-3 post-treatment, respectively. However, noticeable sand fly reduction did not occur after week-3 and results suggest environmental factors also influenced abundance significantly. In conclusion, fipronil bait, applied in southeastern Kazakhstan, has the potential to reduce or potentially eliminate *Xenopsylla* spp. fleas if applied at least every 80-days, but may need to be applied at higher frequency to significantly reduce the oviposition rate of *Phlebotomus* spp. sand flies. Fipronil-based bait may provide a means of controlling blood-feeding vectors, subsequently reducing disease risk, in Central Asia and other affected regions globally.

## Author summary

Plague and cutaneous leishmaniasis are two diseases transmissible to humans vectored by fleas and sand flies, respectively. Although the diseases are vectored by two different insect types, the primary reservoir host for both diseases in desert foci in Central Asia is the great gerbil. Therefore, a promising strategy for controlling both vectors, and subsequently both diseases, is to target the host. A field study was conducted in southeastern Kazakhstan to evaluate the use of a rodent bait containing an insecticide (0.005% fipronil), applied to active gerbil burrows, in reducing field-caught flea and sand fly abundance. Results suggested that the fleas infesting gerbils could be reduced to zero for at least 80-days after completing treatment. The number of reproductive female sand flies were also reduced to zero 3-weeks after completing treatment, but environmental factors also influenced abundance. Additionally, because the bait contained a low insecticide concentration, the application rate remained low, posing reduced risk to non-target animal species. Our approach incorporates detailed evaluation of the use of a systemic insecticide in controlling fleas infesting gerbils and sand flies caught in light traps. Hence, this study provides an explicit means of evaluating the use of a new vector control approach in reducing two distinct insect vectors parasitizing the same host.

## Introduction

Vector-borne diseases transmissible to humans were responsible for more human disease and death than all other causes combined between the 17^th^ and 20^th^ centuries [1] and since the 1970s a global reemergence of several vector borne diseases has occurred [2]. Vector-borne diseases occur most frequently in areas of extreme poverty [3], and cost-effective measures, which consider socio-economic and environmental risk factors are warranted. Vector-borne diseases are prevalent in the Central Asian Republic of Kazakhstan, with plague (*Yersinia pestis*) and cutaneous leishmaniasis (*Leishmania major*) being two of concern [4,5].

### Plague

Plague is a fatal, rodent-associated, flea-borne pathogen found throughout Asia, Africa and the Americas. Between 2010–2015, 584 plague-related deaths were reported globally [6]. While plague-induced mortality is far less common than during the pandemics of previous centuries [7], the disease is still regarded as “emerging” and changes in land-use have increased the probability of interaction between host species and humans [8]. Additionally, plague can wreak havoc on native wildlife, such as in North America, where plague outbreaks damage efforts to re-introduce endangered black-footed ferrets (*Mustela nigripes*) [9] which are dependent on plague-susceptible black-tailed prairie dogs (*Cynomis ludovicianus*) for food and habitat [10]. The last plague outbreak to occur in central Asia occurred in the Qinghai province of China in 2009 in which 12 people tested positive for plague with 3 people dying [11]. Central Asian plague outbreaks have been reduced in recent years with 17 cases and 8 deaths reported 2010–2015 [12]. However, desert plague is still a focus in central Asia because of the increasing risk of plague outbreaks from factors such as anthropogenic influence [13] and climate change [14].

### Cutaneous leishmaniasis

Cutaneous leishmaniasis (CL) is a sand fly-borne neglected tropical disease. Although not lethal, it results in ulcerations on the skin which can lead to severe disability and lifelong scars, often resulting in severe social prejudice [15]. It is by far the most common form of *Leishmania* with ~700000–1200000 new cases being reported annually [16]. Although primarily a disease of the poor, as of 2009 it was estimated that there were ~3000–5000 cases of CL among U.S. military personnel [17]. As a result, the Deployed Warfighter Protection Research Program (DWFP) has invested in developing new pesticides for sand fly control [18]. The disease is difficult to control in part because aspects of sand fly ecology remain largely unknown [19]. In Central Asia, zoonotic CL is largely rodent associated [20]. Less than 100 cases were reported in Kazakhstan in 2015 [21] and of 333 leishmaniasis cases reported in Kazakhstan between 1996–2006, 332 of them were zoonotic CL [22].

### The great gerbil

The Great gerbil (*Rhombomys opimus*) is a colonial rodent that is considered the primary reservoir of plague [23] and zoonotic CL [24] in Central Asia and Kazakhstan in particular. In most instances, human plague infestation occurs because of epizootics among wild rodent populations, while the sources of infection are linked most closely with fleas and less often with contact with wild animals [25]. Great gerbils live in family groups that inhabit and defend burrow systems (gerbil colonies) [26]. These gerbil colonies are extensive, typically ranging from 15–40 m in diameter [27], but the diameter of a single colony can exceed 50 m with the sizes of colonies dependent on the nature of the soil and vegetation cover [28]. These complex and usually well-marked structures have a pronounced ecological center and periphery, with up to several hundred burrow entrances [29]. The total length of underground passages is on average 300–400 m but will occasionally exceed 1 km [30]. The depth of burrow systems generally averages 2–3 m [30]. The burrows of great gerbils play an important role in desert ecosystems of Central Asia and Kazakhstan because many animal species are associated specifically with the burrows of these rodents [31].

The number, position and size of gerbil colonies do not change over time, but the occupancy of these gerbil colonies can fluctuate greatly, and disease abundance fluctuates in response [32]. The rodent is regarded as an enzootic host for plague, in that the infections have been reported in high seroprevalence, but mass mortalities of gerbils are not often reported [23]. The vectors of plague and CL in the system involving great gerbils are *Xenopsylla* spp. fleas [32], particularly *X*. *gerbili minax* [33,34], and *Phlebotomus* spp. phlebotomine sand flies [35,36], respectively.

### Vector control

Currently, indoor residual spraying (IRS) is one of the primary measures for control of endophagic flying vectors such as mosquitoes and phlebotomine sand flies [37]. Because the success of this type of control is dependent on vectors being endophilic [37], IRS may not be a logical means of control in desert-type areas where sand flies inhabit burrows. Additionally, recent research suggests that current program-initiated IRS control may not adequately reduce sand fly abundance in areas where they are believed to be primarily endophagic and endophilic [38]. A common technique for controlling ectoparasite vectors such as ticks and fleas in burrow systems is to dust rodent burrows with insecticides such as deltamethrin [39,40] or permethrin [41], and this is the preferred method of controlling arthropod pests such as fleas and sand flies inhabiting great gerbil burrow systems in Kazakhstan [25]. Dusting is reported to be costly and have negative impacts on non-target species [42]. Fleas and sand flies have both previously been reported to be resistant to insecticides used in IRS and insecticide dusting campaigns [43,44], because of the large amount of insecticide which is required to perform these applications.

Systemic insecticides (endectocides) could provide an additional means of controlling disease vectors which blood feed on desert rodent species, by directly targeting the host with a bait containing a reduced insecticide concentration. The phenylpyrazole, fipronil, is a broad spectrum insecticide which disrupts the central nervous system of insects [45]. The whole blood half-life of a single oral dose (4 mg/kg) administered to rats is 6.2–8.3 days [46] and it is liposoluble which results in prolonged insecticidal effect in organisms [47]. Approximately 45–75% of fipronil may be excreted in rodent feces and 5–25% in the urine [46]. The excretion of fipronil in rodent feces may be beneficial in larval control. Fipronil has been shown to be highly efficacious against *Phlebotomus argentipes* sand fly adults and larvae when administered to lesser bandicoot rats (*Bandicota bengalensis*) and roof rats (*Rattus rattus*) [48]. It has also proved effective against *P*. *papatasi* feeding on *Meriones shawi* under laboratory and field conditions in Tunisia [49]. Additionally, pen and field studies suggest that phlebotomine sand flies and *Anopheles* spp. mosquitoes blood feeding on fipronil-treated cattle (*Bos taurus*, *Bos indicus*) can be reduced significantly [50–52]. Fipronil administered to rodents has also been highly efficacious in reducing on-host ectoparasites such as *Ixodes* spp. ticks [53,54] and *Oropsylla* spp. [55] and *Xenopsylla* spp. [56] fleas. Recently, a rodent grain bait (0.005% fipronil) applied to the openings of black-tailed prairie dog burrows, at a rate of 0.096 mg fipronil/m^2^, resulted in >95% reduction in on-host fleas infesting prairie dogs for a minimum of 52 days post-treatment application [55]. The potential for a fipronil-based bait to successfully target fleas and sand flies feeding on rodents has been indicated by these previous experiments. However, the impact of a rodent grain bait (0.005% fipronil) on *Xenopyslla* spp. flea and *Phlebotomus* spp. sand fly abundance, has never been reported in a field trial in southeastern Kazakhstan or anywhere else in Central Asia.

### Objectives

The objectives of this study were to 1) apply 0.005% fipronil bait to active great gerbil burrows in southeastern Kazakhstan and 2) monitor *Xenopyslla* spp. flea and *Phlebotomus spp*. sand fly abundance. The great gerbil is the primary reservoir of plague and zoonotic CL in Central Asia and therefore serves as an abundant blood meal source for fleas [39] and phlebotomine sand flies [24]. A reduction in flea and sand fly abundance could potentially lead to a reduction in risk of human plague and CL transmission. The results of this study will help determine the efficacy of a fipronil bait in controlling fleas and phlebotomine sand flies when targeting a host upon which both blood feed.

## Materials and methods

### Ethics statement

All activities involving animals for this study were reviewed and approved by the Institute of Animal Care and Use (IACUC) at Genesis Laboratories and followed the Animal Welfare Act and Genesis Laboratories Animal Care and Use Guidelines (Approval Date March 18, 2016).

Additional approval for animal use was granted by the Animal Care and Use Review Office (ACURO) of the US Army Medical Research and Material Command (Approval for Protocol No. CBMS-FY15-010.03 Dated: April 29, 2016).

The authors whose affiliations were with the M. Aikimbaev’s Kazakh Science Centre for Quarantine of Zoonotic Diseases were the only individuals who trapped gerbils in the field. Animals regarded as pest species (such as the great gerbil) do not require permits for trapping within the territory surveyed in southeastern Kazakhstan.

### Study area

The study was conducted in southeastern Kazakhstan (N 44.93621 E 76.02039), ~200 km north of Almaty city and 24 km northwest of Bakanas (June 1-September 3, 2016). The specific timing of study events are available in S1 Table. The Ili River, a major source of moisture in the region [57], was located ~16 km west. The study area was northern subzone desert, composed of flood plains and dunes, and consisting mainly of sandy and clay soils, with saxaul (*Haloxylon aphyllum* and *H*. *persicum*) being the primary vegetation.

**Fig 1:**
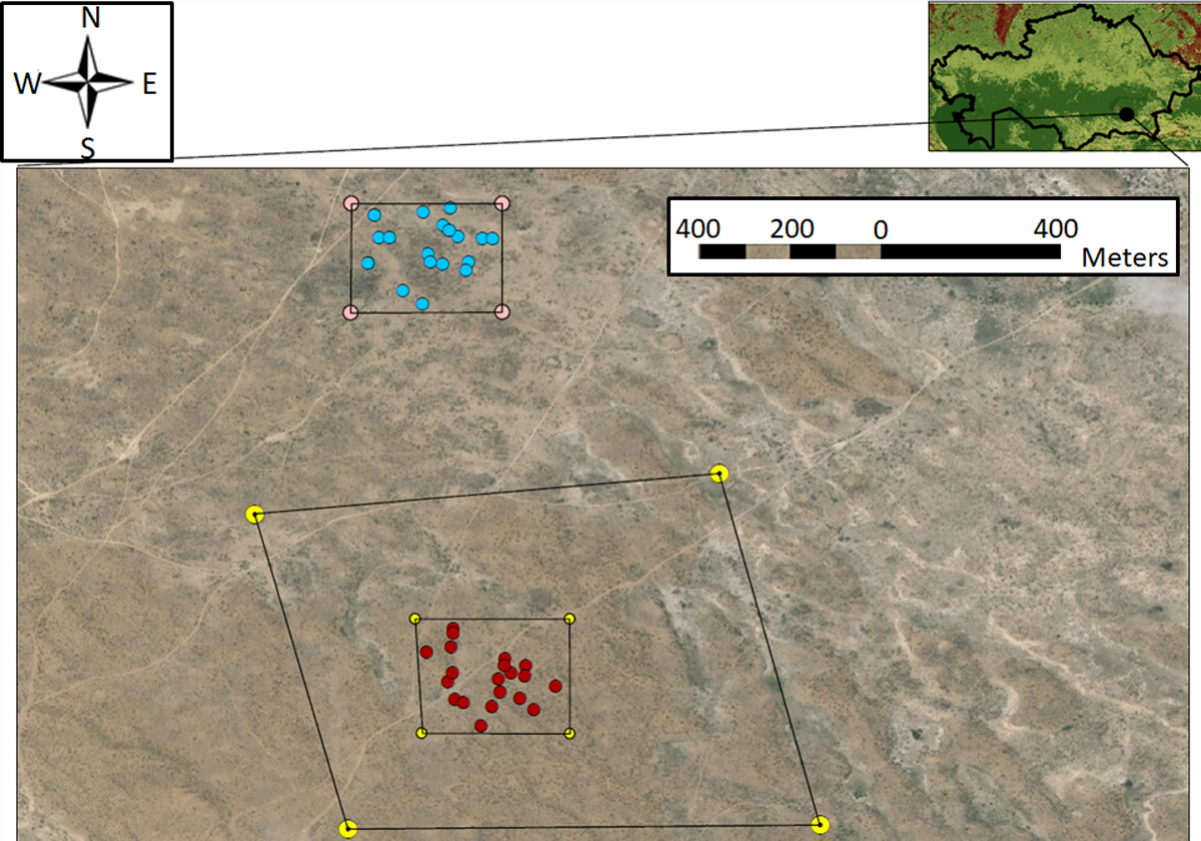
Map of boundaries and gerbil colony locations with the treatment (bottom) and control (top) plots. Red and blue dots indicate selected colony locations within the treatment and control plots, respectively. Small squares surrounding the treatment and control plots indicate the plot boundaries. The larger boundary surrounding the treatment plot indicates the buffer zone. Map was generated in ArcGIS using ArcMap with a World Imagery base layer (Source: Esri, DigitalGlobe, GeoEye, Earthstar Geographics, CNES/Airbus DS, USDA, USGS, AeroGRID, IGN, and the GIS User Community).

Two test areas were selected, one where 0.005% fipronil bait was applied to all active burrows (Treatment); and one where all active gerbil burrows remained untreated (Control). The boundaries of the test areas were separated by >400 m. The “Treatment area” was comprised of 1) a ~78750 m^2^ “Treatment plot”, from which flea and sand fly sampling would be performed, and 2) a “Treatment buffer zone”, extending ~200–600 m from the Treatment plot perimeter, which was established to account for gerbil movement, which has been estimated to be <200 m for >95% of individuals [58]. The “Control area” consisted only of a ~78750 m^2^ “Control plot” and no buffer zone was needed because no bait was applied. The main criterion for plot selection was the presence of >20 great gerbil colonies within each test plot. Trap sites were selected within the Treatment plot (*n* = 20) and Control plot (*n* = 20). GPS coordinates (Garmin Etrex 30, Olathe, KS, USA) were taken for all trap sites and the corners of the Treatment plot, Control plot and Treatment buffer zone (Fig 1). Environmental data (temperature, humidity, precipitation, wind speed) were collected from the nearest accessible monitoring station in Bakanas [59].

### Occupancy status

A gerbil colony census was conducted to determine the total number of gerbil colonies within the study areas, and their individual occupancy status (occupied, visited, empty). “Occupied” burrow systems were defined as those occupied by a family group. “Visited” colonies showed signs of activity, such as food storage, but were not occupied by a family group [27]. “Empty” colonies were those which had been completely deserted. All gerbil colonies were mapped using a handheld GPS (Garmin Etrex 30, Olathe, KS, USA).

### Rodent grain bait (0.005% fipronil)

#### Bait preparation

The rodent grain bait, containing a nominal concentration of 0.005% fipronil (50 mg/kg), was prepared locally in Almaty, Kazakhstan, using a locally purchased cement mixer, and modified methods described by [55]. To confirm fipronil concentration in the bait prior to field application, a validated method of High-Performance Liquid Chromatography (HPLC) reverse phase with UV detection was used. The mean fipronil concentration in the bait was determined to be 51.1 mg/kg (*n* = 4, recovery = 102%).

#### Bait acceptance

**Fig 2:**
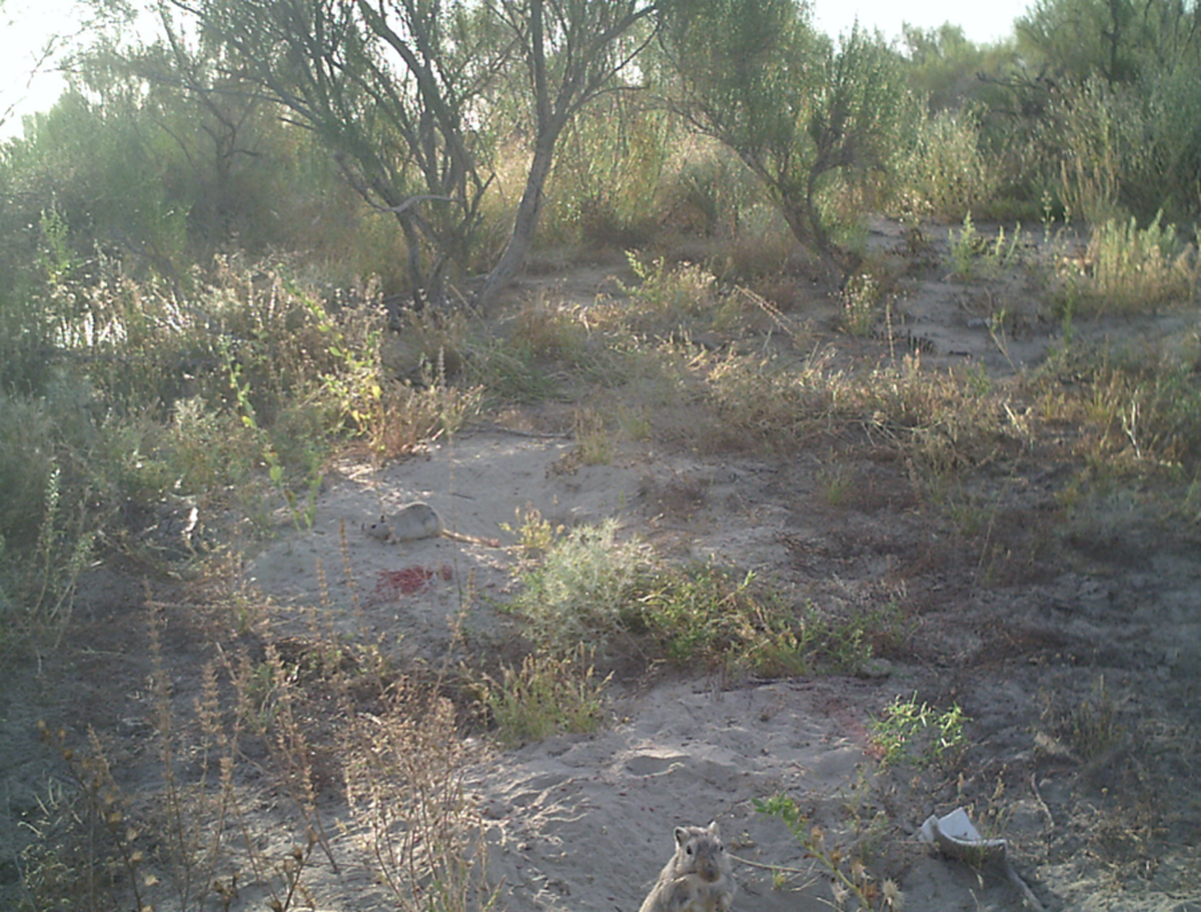
Adult great gerbils (*Rhombomys opimus*) consuming fipronil-based grain bait (red).

To ensure bait uptake by great gerbils prior to field application, ~120 g bait was applied within 1 m of four active gerbil burrows a considerable distance from the test areas (~5 km). Two digital trail cameras (Primos: Truth Cam 35) were mounted ~3 m from treated burrows to capture video and photo evidence of gerbils consuming bait. All bait was consumed by gerbils within 24 hours. Video footage (S1 Video) and photo images (Fig 2) confirmed uptake of the bait by great gerbils.

### Pre-treatment

Prior to application of fipronil bait, field-collected sand flies and fleas were used to estimate baseline abundance for 2 weeks (June 1-June 15).

#### Gerbil live trapping and flea collection

**Fig 3:**
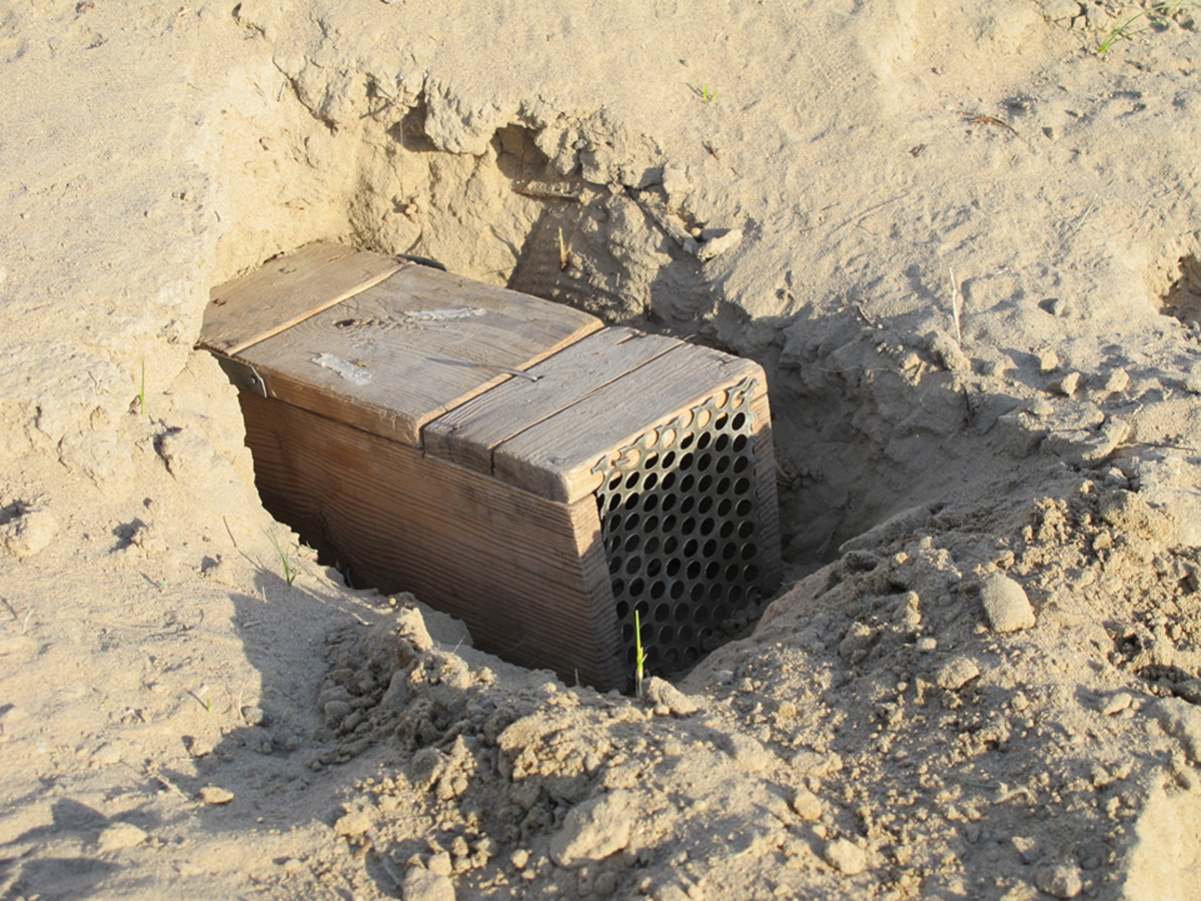
A wooden great gerbil trap positioned in the entrance of an active gerbil burrow.

The on-host flea population was estimated by live-trapping gerbils and combing their fur. Gerbils were trapped, at the designated trap sites within each test plot (*n* = 20 per plot), using locally-manufactured, wooden Zaycev live-traps (~7.6H x 7.6W x 25L cm). The open end of each trap was inserted into the opening of an active gerbil burrow. The closed end of each trap was vented to allow sunlight in, making it appear that the burrow opening was not obstructed (Fig 3). Traps were not baited. Gerbil trapping, and flea collection were conducted 1-3x per week dependent on trapping success and availability of trapping personnel. On average 10–20 traps were set within each test plot during each trapping period.

Captured gerbils were processed using methods similar to those described by [60]. Gerbils were identified by gender and age (juvenile or adult). Gerbils were then restrained over a porcelain container and all animal fur was brushed with a flea comb. Recovered fleas were then collected with a hand-pump aspirator. Fleas were then transferred into 50 ml conical tubes labelled to correspond with trap location and date of collection. Gerbils were uniquely marked using permanent ink to ensure that individuals would not be recaptured within the same trapping period. Additionally, trapping was not performed more than once at an individual trap sight within each trapping period. After all fleas were collected, gerbils were immediately released at the point of capture. Recaptured individuals were immediately released without combing. Because no blood collection or pain-inducing marking procedures were used, gerbils were not anesthetized during flea collection. Trapping success varied, but the goal of the experiment was to sample at least 20 great gerbils within each plot.

#### Sand fly collection

Sand flies were captured using Centers for Disease Control and Prevention (CDC) light traps (John W. Hock Company, Gainesville, FL, USA), which have proven effective in collecting sand flies during prior field studies [61–63]. CDC light traps were secured to adjacent vegetation at trap sites, vertically ~1 m above the ground, and fitted with a protective cover to shield the mechanical components from rain and falling debris [63]. Within each plot, 20 CDC light traps (1 trap/trap site; 40 CDC light traps total) were activated at ~18:00 and collected at ~06:00. Trap catches were returned to the laboratory and stored in a -20°C freezer. CDC light traps were set and collected at least weekly, 17 times over ~8-weeks. Trapping events were typically separated by 3–5 days. However, if weather conditions reduced sand fly abundance in traps within both plots (<2 sand flies per trap), trapping would be performed over consecutive nights (S1 Table). Because dry-ice was not available within the town of Bakanas, we were unable to compare the efficiency CDC light traps with CO_2_ traps, another validated method of sand fly collection [64].

#### Flea identification

Fleas were counted, sexed, and morphologically identified by species using methods described by [65]. Fleas were placed into 50 ml conical tubes labelled to correspond with trap location, date of collection, age and sex of the gerbil captured, and the number of fleas by sex and by species.

#### Sand fly identification

Phlebotomine sand flies were counted, sexed, and morphologically identified by genus (*Phlebotomus* spp., *Sergentomyia* spp.). Specimens were placed into petri dishes labelled to correspond with trap location, date of collection, and the number of sand flies by sex, species and reproductive status (gravid, non-gravid).

Because of morphological similarities between certain *Phlebotomus* spp. sand flies in Central Asia [66] species confirmation was a multi-step process. *Phlebotomus* spp. sand flies were dissected by sterile micro-needles. Heads and terminal parts of abdomens bearing distinctive morphological characteristics (pharyngeal armature, cibarium, genitalia) were mounted in CMCP-10 medium (Polysciences, Germany) and identified using a morphological key [67]. The identity of chosen specimens was further confirmed by sequencing a fragment of cytochrome B mitochondrial gene. Genomic DNA was isolated by High Pure PCR Template Preparation Kit (Roche, France) following the manufacturer’s instructions. Primers and PCR conditions previously described by [68] were applied. The amplification products were separated and visualised on 2% agarose gels, purified using the QIAquick PCR Purification Kit (Qiagen) and directly sequenced in both directions using the primers used for DNA amplification (ABI Prism BigDye Terminator Cycle Sequencing Ready Reaction Kit, USA). Obtained sequences were compared with those deposited in GeneBank using online nucleotide BLAST tool.

### Treatment

At treatment Day-0 (June 16) ~120 g of fipronil bait was applied <1 m from each active gerbil burrow of each occupied and visited gerbil colony within the boundaries of the treatment area. Fipronil bait was effectively applied to ~771908 m^2^. A second analogous treatment application was performed June 21. Vector collection continued to be conducted as was done during the pre-treatment study period (June 16-June 21).

### Post-treatment

Flea and sand fly collection continued to be conducted as was done during the previous study periods (Post-Treatment 1: June 22-July 29). Gerbil trapping, and flea collection were performed again at Study Days 79–80 (Post-Treatment 2: September 3–4) because prior studies have indicated systemic insecticides to suppress flea abundance by up to ~94% at 2-months and up to ~88.5% at 3-months post-treatment [69].

### Non-target vertebrate species

During the treatment and post-treatment periods (June 16-September 4), visual observations were performed 4x/week within the treatment area to monitor for any unexpected abnormal animal behavior, primary and secondary non-target mortality, or other negative signs that could be associated with fipronil bait application. The treatment area was traversed on foot and live animals (gerbils, foxes, etc.) were observed from a distance (>50 m) using binoculars. Any change in animal appearance was recorded.

### Animal welfare

All animal activities performed during this study followed Animal Welfare Act regulations and were approved by the Genesis Laboratories, Inc. Institutional Animal Care and Use Committee (USDA Animal Welfare Act, 9 CFR Parts 1–3) and the Animal Care and Use Office of the US Army Medical Research and Material Command. Gerbil trapping was performed by researchers employed by the M. Aikimbaev’s Kazakh Science Centre for Quarantine of Zoonotic Diseases (Almaty, Kazakhstan), who had permission to perform the live-trapping.

### Data analyses

Infested great gerbils were defined as individuals having a minimum of one *Xenopsylla* spp. flea. The efficacy of fipronil bait was estimated by calculating flea and sand fly indices. The flea index was defined as the mean number of *Xenopsylla* spp. fleas collected per captured gerbil per plot during a single sampling period (Pre-treatment, Treatment, Post-treatment). The sand fly index was defined as the mean number of non-gravid female and gravid female *Phlebotomu*s spp. sand flies collected per trap-night per plot during a single sampling period. Trap-night is defined as the average number of sand flies collected per trap per night of trapping. Male sand flies do not blood feed, feeding exclusively on sugar from plants [70] and hence do not transmit CL. Therefore, they were not used to calculate efficacy. Most female sand flies are gonotrophically concordant [71], requiring one blood meal for each batch of eggs produced, and reduction in gravid female sand flies could indicate a decrease in the rate of reproduction due to the ingestion of a blood meal acquired from a fipronil treated host. Pre-treatment, treatment, and post-treatment mean sand fly and flea indices were used to calculate efficacy of rodent grain bait (0.005% fipronil) in reducing vector abundance. For fleas, efficacy was determined by comparing the Pre-treatment flea index with the flea indices calculated during Treatment, Post-treatment 1, and Post-Treatment 2. For sand flies, abundance fluctuated dramatically during each time point and therefore efficacy was determined weekly during the post-treatment period. the Pre-treatment sand fly index was compared with indices calculated during Treatment, Post-Treatment Week-1 (PTW-1), PTW-2, PTW-3, PTW-4, PTW-5, and PTW-6. Efficacy of fipronil in reducing flea and sand fly indices was adjusted for potential vector reduction within the control plot using an equation described by [72].

Nonparametric statistical methods were used to determine significant differences in relative abundance (*p*<0.05). Differences in vector abundance occurring during pre-treatment, treatment, and post-treatment were compared between and within the study plots using a Wilcoxon Rank Sum test. Differences in nightly changes (+) in vector abundance in treatment and control were evaluated using a sign-test, to determine whether treatment might influence the tendency for vector abundance in increase and decrease during each trapping period.

## Results

Environmental conditions in Bakanas were reported June-September 2016 (S2 Table). July was the warmest month (mean: 25.7°C) and September the coldest (mean: 19.7°C). Humidity was highly variable ranging from 6–100%. The greatest precipitation occurred in June (49.6 mm) decreasing exponentially in the months to follow, the least precipitation occurring in September (5.1 mm). The highest wind speed reported was in August (36 km/h) followed by June (32 km/h), the averages being 12.7 and 10.9 km/h, respectively.

### Occupancy status

The occupancy status of individual gerbil colonies was obtained from 156 colonies within the treatment area (buffer = 127 + inner plot = 29) and from 31 colonies within the control plot. Gerbils occupied the majority of the burrow systems surveyed within the treatment (77%) and control areas (68%). Approximately 9% of the total colonies surveyed were “visited” with the remainder being “empty”.

### Fipronil bait application

**Fig 4:**
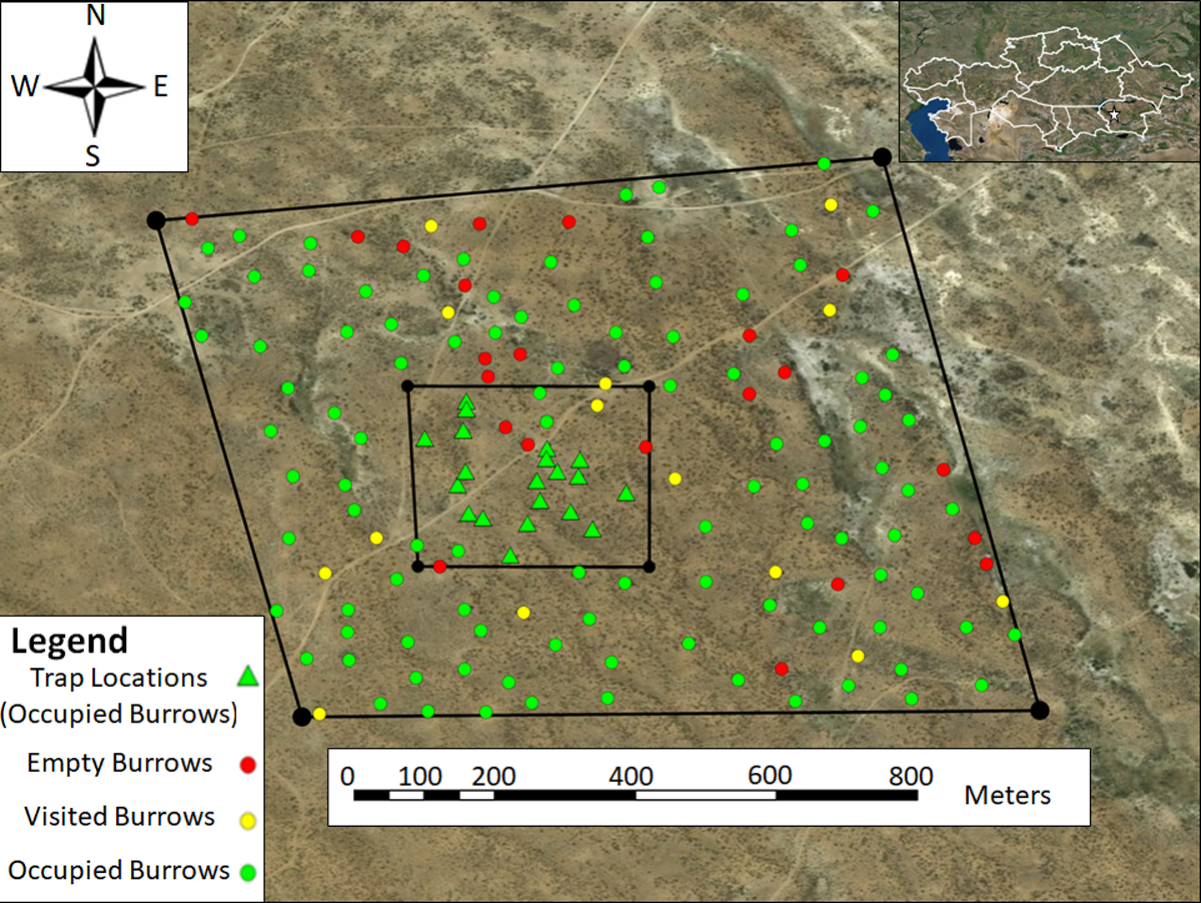
The location and occupancy status of all 134 gerbil colonies within the treatment area upon which two fipronil bait applications were performed June 16 and June 21, 2016. Map was generated in ArcGIS using ArcMap with a World Imagery base layer (Source: Esri, DigitalGlobe, GeoEye, Earthstar Geographics, CNES/Airbus DS, USDA, USGS, AeroGRID, IGN, and the GIS User Community).

During each bait application, a total of 100 kg bait (~5 g fipronil) was used to treat active gerbil burrows of 120 occupied and 14 visited great gerbil colonies within the ~771908 m^2^ treatment area (Fig 4). Considering the treatment plot area (~771908 m^2^) and number of colonies treated (*n* = 134), this amounted to 129.5 mg bait/m^2^ and 0.006 mg fipronil/m^2^ being applied during this study (Table 1).

**Table 1:** Bait and fipronil application rates per **(A)** total treatment area; and **(B)** treated colony.

**A** Treatment No. (Date Applied)	Bait/Application (kg)	Area Treated (m^2^)	Bait Application Rate (mg/m^2^)	Fipronil Application Rate (mg/m^2^)
Treatment 1 (June 16)	100	771908	129.5	0.006
Treatment 2 (June 21)				
Total	200	-	-	-
**B** Treatment No. (Date Applied)	Bait/Application (kg)	No. Colonies Treated	Bait Application Rate (g/colony)	Fipronil Application Rate (mg/colony)
Treatment 1 (June 16)	100	134	746	37
Treatment 2 (June 21)				
Total	200	-	-	-

### Vector species identification

#### Fleas

Fleas were collected from a total of 56 wild great gerbils (42 adults, 14 juveniles) captured within the Treatment (*n* = 25) and Control (*n* = 31) plots. A total of 385 fleas, of which 379 were *Xenopsylla gerbili minax* (>98%), were removed from the gerbils and morphologically identified over the course of the experiment (Tables 2 and S3). Sixty-six-point one percent (66.1%) of *X*. *gerbili minax* were female.

**Table 2:** *Xenopsylla gerbili minax* fleas collected within the treatment and control plots from wild-caught great gerbils during the pre-treatment, treatment, and post-treatment periods.

Plot ID	Study Period: Study Days (Date Range)	Collected *Xenopsylla gerbili minax*		
		Total	Male	Female
Treatment	Pre-treatment (June 1-June 15)	19	6	13
	Treatment: Day 0-Day 5 (June 16-June 21)	0	0	0
	Post-treatment 1–2 Day 6-Day 80 (June 22-September 4)	0	0	0
	Total	19	6	13
Control	Pre-treatment (June 1-June 15)	31	12	19
	Treatment: Day 0-Day 5 (June 16-June 21)	77	17	60
	Post-treatment 1–2 Day 6-Day 80 (June 22-September 4)	252	93	159
	Total	360	122	238

#### Sand flies

In total, 9555 sand flies were captured over 533 total trap-nights (S4 Table). Ninety-seven-point eight percent (97.8%) of the specimens were determined to be *Phlebotomus* spp. with (55.6%) being female (Table 3). Seventeen-point seven percent (17.7%) of the *Phlebotomus* spp. females were gravid. All *Phlebotomus* spp. sand flies identified using the previously described methods [68–69] were *P*. *mongolensis*.

**Table 3:** Total *Phlebotomus* spp. sand flies collected in CDC light traps set 17 times from June 1-July 29, 2016.

Plot ID	No. Trap-nights	*Phlebotomus mongolensis*			
		Total	Male	Non-gravid Female	Gravid Female
Treatment	267	3965	1757	1881	327
Control	266	5378	2386	2401	591
Total	533	9343	4143	4282	918

### Non-target vertebrate species

No dead or moribund gerbils or non-target animals were observed within or around the treatment area. Additionally, no abnormal animal behavior was observed during the study. However, gerbils within the treated area appeared to have healthier skin and fur (reduced mange, sores) than those within the control area.

### Fipronil bait efficacy

#### Fleas

Fipronil-based bait application was efficacious against *X*. *gerbili minax* infesting great gerbils, resulting in 100% reduction for a minimum of 80 days (Tables 2 and 4). These results exceed the >90% efficacy recommended by the EPA for flea control [73].

**Table 4:** The efficacy of bait (0.005% fipronil) in reducing the flea index of *Xenopsylla gerbili minax*.

Study Period: Study Days (Date Range)	Control			Treatment			
	No. Gerbils Sampled	Infestation Rate (%)	Flea Index^1^ (+SD)	No. Gerbils Sampled	Infestation Rate (%)	Flea Index^1^ (+SD)	Efficacy (%)
Pre-treatment (June 1-June 15)	6	100	5.2 (+6.3)	6	100	3.2 (+3.1)	-
Treatment: Day 0-Day 5 (June 16-June21)	7	100	11 (+7.5)	5	0	0	100*
Post-treatment 1: Day 6-Day 43 (June 22-July 29)	14	100	16.9 (+10.8)	10	0	0	100*
Post-treatment 2: Day 79-Day 80 (September 3-September 4)	4	100	3.8 (+3.3)	4	0	0	100*

^1^Flea Index = mean number of *Xenopsylla gerbili minax* per captured gerbil.

*Exceeds recommended flea efficacy of >90% [73]

Differences in the on-host flea indices between treatment and control were significant during treatment (*p* = 0.0041) and post-treatment (*p* = 0.0001) but not during pre-treatment (*p* = 0.6809). Flea abundance during the pre-treatment, treatment and post treatment periods did not differ significantly within the control plot (*p* = 0.2051). Flea abundance was significantly different within the treatment plot (*p*<0.0001) between pre-treatment and treatment (*p*<0.0053) and post-treatment (*p*<0.0001).

#### Sand flies

Efficacy of bait in reducing non-gravid female *P*. *mongolensis* abundance reached a maximum of 18.7%, occurring PTW-1 (Table 5). The bait appeared more efficacious against gravid *P*. *mongolensis*, which were reduced by ~72% during treatment exceeding the requirements of a high-efficacy product (70–90%) [74], and were reduced by 100% during PTW-3, which exceeds the 95% efficacy recommended by [75]. However, the gravid female abundances within both test areas were low in comparison to general relative sand fly abundance. Sand fly numbers were not noticeably reduced after PTW-3. Sample collection was terminated after PTW-6.

**Table 5:** The average number of sand flies per trap-night (+SD), efficacy of bait (0.005% fipronil) against female *Phlebotomus mongolensis* sand flies (non-gravid and gravid), and average minimum temperature, maximum humidity, and precipitation (+SD) recorded during all study periods.

Study Period	Sand Fly Index: Control		Sand Fly Index: Treatment		Efficacy (%)^1^		Environmental Conditions		
	Non-gravid	Gravid	Non-gravid	Gravid	Non-gravid	Gravid	Min Temp (°C)	Max Wind Speed (km/h)	Precipitation (mm)
Pre-treatment	16.7 (+19.5)	2.1 (+3.4)	13.0 (+16.6)	1.4 (+2.6)	-	-	18 (+0.7)	12.5 (+8.1)	0.9 (+1.2)
Treatment	3.5 (+4.7)	0.5 (+1.2)	3.3 (+3.2)	0.1 (+0.3)	0	72.0 *	16.5 (+3.5)	9 (+2.0)	1.5 (+1.5)
^2^PTW-1	4.0 (+5.1)	0.8 (+1.7)	2.5 (+3.7)	0.4 (+0.9)	18.7	23.8	17 (+3.6)	17.7 (+10.5)	3 (+0)
PTW-2	14.2 (+17.7)	8.5 (+9.0)	10.2 (+10.5)	4.4 (+7.1)	8.1	22.8	20.5 (+0.5)	10.5 (+3.5)	0.1 (+0.1)
PTW-3	4.0 (+3.0)	0.3 (+0.5)	3.4 (+2.8)	0 (+0)	0	100**	21 (+0)	7 (+0)	0 (+0)
PTW-4	3.6 (+4.6)	0.4 (+1.1)	5.1 (+7.2)	0.3 (+0.8)	0	0	20.3 (+1.2)	19.3 (+6.0)	0 (+0)
PTW-5	0.4 (+0.5)	0 (+0)	1.0 (+0.6)	0 (+0)	0	0	19 (+0)	0 (+0)	4 (+0)
PTW-6	0.1 (+0.3)	0.1 (+0.3)	1.5 (+1.9)	0.4 (+0.9)	0	0	20 (+0)	14 (+0)	2 (+0)

^1^Efficacy calculated using the method described by [72].

^2^PTW = Post-treatment Week

*Qualifies as high-efficacy vector control technology (70–90%) [74]

**Exceeds recommended efficacy of >95% [75]

*P*. *mongolensis* gravid female abundance within the treatment area was significantly lower than that of the control during treatment (*p* = 0.035) and post-treatment (*p* = 0.0026) but not during pre-treatment (*p* = 0.5585). Significant differences were detected between study periods within the treatment plot (*p* = 0.0251) with the most significant gravid sand fly reduction occurring between pre-treatment and treatment (*p* = 0.0076). However, gravid sand fly numbers also differed significantly between pre- and post-treatment within the control plot (*p* = 0.0314), which would indicate that environmental variables could have markedly influenced sand fly abundance. Environmental variables such as minimum temperature [76–79], maximum windspeed [79,80], and precipitation [78,80] have influence on sand fly abundance. Sand fly abundance differed significantly at variable temperatures (°C), windspeed (km/h), and precipitation (mm) (Wilcoxon: *p*<0.0001). This might help explain the decrease in efficacy observed during PTW-1 and PTW-2, during which increases in temperature, windspeed, and precipitation occurred (Table 5). Results of a nonparametric sign test suggested that nightly changes in sand fly abundance (+) between the treatment and control plot did not differ significantly (*p* = 0.0654) during the study.

## Discussion

The results of this study suggest that fipronil bait, applied twice during a 5-day treatment period, at a rate of ~0.006 mg fipronil/m^2^, 1) may be of reduced risk to great gerbils and non-target wildlife, 2) can significantly reduce or eliminate *Xenopsylla* spp. fleas for at least 80 days post-bait application, and 3) shows inconsistent efficacy against female *Phlebotomus* spp. sand flies, suggesting that different methodology such as more frequent applications may need to be implemented. To our knowledge, this is the only field trial, evaluating the use of a fipronil-based rodent bait in controlling fleas and sand flies, to be conducted in Central Asia.

No *Xenopsylla* spp. fleas were collected within the treatment plot after bait application, suggesting that 100% control can be maintained for a minimum of 80 days post-treatment when applied in mid-June. Additionally, fleas were collected from the openings of active gerbil burrows, using handpump aspirators, five times during post-treatment between July 1 and July 29 within the Treatment and Control plot. No *X*. *gerbili minax* were collected from burrows within the Treatment plot while multiple (*n* = 212) were collected within the Control plot. The lack of baseline data for burrow fleas prohibited data analyses. However, the complete absence of *X*. *gerbili minax* from the sampled burrow entrances is worth noting, given the 0% *X*. *gerbili minax* infestation rate amongst Treatment plot gerbils relative to the 100% infestation rate in Control plot gerbils observed throughout the study. Considering the inability of fleas to survive outside of the burrow systems [22] our results would suggest that seasonal fipronil bait application could potentially remove *Xenopsylla* spp. fleas from plague-endemic areas. We should note that a single flea, of a different genus (*Coptopsylla lamellifer*) [81], a moderate plague vector [82], was collected within the treatment plot during September, suggesting that a second autumn treatment might be beneficial. In the future, studies should be designed to establish a baseline for burrow fleas during the pre-treatment period to better estimate the efficacy of the bait.

Although treatment against fleas was successful, the precise rate of decline in efficacy of the bait was not calculable because 100% efficacy was still being achieved at test termination. [69] saw up to 90-day efficacy of imidacloprid-based bait in reducing fleas infesting ground squirrels and [48] found fipronil to be superior to imidacloprid for sand fly control. [55] reported fipronil efficacy of >90% against fleas infesting prairie dogs for at least 52-days post-initial application. Knowing the length of time required to achieve significant flea reduction would be highly beneficial to managers and might further suggest that two treatments performed in spring or autumn might be sufficient to reduce flea infestations.

Fipronil efficacy against gravid sand flies was >70% during the treatment period and up to 100% during PTW-3, with reduced efficacy observed afterwards. Although gravid females were reduced markedly during these periods, 1) their relative abundance was low when compared with general sand fly abundance, 2) sand fly abundance differed significantly during the pre-treatment, treatment, and post-treatment periods within each test plot, and 3) fluctuations in relative abundance did not differ significantly between plots. Field populations of sand flies are sensitive to climatic variables such as temperature, strong winds and heavy rain [83]. The reduced efficacy during PTW-2 and PTW-3 may have been a result of the increase in windspeed. Several researchers have studied the movement of *Phlebotomus* spp. sand flies and have suggested that although they typically fly short distances, they may occasionally move distances more than 1–2 km [64,83,84]. If *P*. *mongolensis* is capable of flying distances >1 km with wind assistance, then it would suggest that reinvasion of the plots by gravid females may have occurred. If future studies indicate that this a persistent issue that needs to be addressed, future studies may consider treating more frequently and/or increasing the size of the buffer zone to better account for sand fly movement. However, the logistics of conducting a study within such a large area will need to be carefully considered. Sand fly abundance during PTW-5 and PTW-6 decreased markedly within both plots, possibly in response to increase in precipitation. Although not a subject of this paper, future studies might focus on explicitly evaluating the flight potential of sand flies and the influence of various climatic variables on sand fly abundance in southeastern Kazakhstan.

Several additional ecological factors may have been responsible for the shorter duration and inconsistency of sand fly efficacy. Fleas are ectoparasites that rely heavily on the host they infest and cannot survive outside of the burrow systems [32]. Sand flies are less host-dependent in that only adult female sand flies blood feed and all other life stages (eggs, larvae, pupae) develop in organic matter. Male sand flies do not blood feed and instead feed exclusively on sugars from plants [70] and therefore were not exposed to the bait. Given development from egg-adult can range from 4-weeks to several months dependent on temperature [76], many developing sand flies may have not been exposed to fipronil-treated gerbils during the peak in fipronil blood concentration when efficacy is at its highest, and many sand flies likely emerged as the fipronil concentration in blood declined. This might explain why reduced sand fly efficacy was seen after PTW-3 and would suggest that monthly fipronil application might be a better means of controlling phlebotomine sand flies under these field conditions. Gravid females prefer to oviposit their eggs in areas containing organic material [85]. Previous researchers have shown fipronil excreted in animal feces to be highly efficacious in reducing laboratory reared phlebotomine sand fly larvae [48–50,86]. However, while many researchers have studied the potential oviposition sites of sand flies, there is a deficit of information regarding natural oviposition sites [87] and attempts to collect immature sand flies from the field have proven difficult and unproductive. For example, a researcher in Sudan processed ~2500 kg of soil to recover only a single sand fly larva [88]. Therefore, while it is speculated that sand flies may oviposit near animal feces, we cannot say with certainty that sand fly larvae were exposed to great gerbil feces. While this fell outside of the scope of our study, investigations of the natural oviposition locations of phlebotomine sand flies should be continue to be pursued, as this knowledge would greatly improve sand fly control programs. Managers must consider differences in ecology of fleas and phlebotomine sand flies and establish a clear distinction between the methodology for controlling these respective vectors of plague and CL.

A concern associated with field application of insecticides is possible resistance of the target vector. The cat flea (*Ctenocephalides felis*), is resistant to multiple insecticides [89], but [90] reported no resistance in strains of cat fleas exposed to topical fipronil. We are confident that great gerbils consumed all of the fipronil bait immediately, without storing it in food chambers. We base this assertion on the fact that these large gerbils do not possess cheek pouches like hamsters [91]. The still images and videos taken with trail cameras further support the belief that gerbils consumed the bait. If great gerbils consumed all bait within a single feeding this would lessen the risk of potential insecticidal resistance. However, during future field applications, surveillance of the flea population in treated areas should be conducted to monitor possible insecticide resistance [92].

Occupancy status of gerbil burrows fluctuates rapidly [32] and it seems intuitive that fipronil-based treatment would be most appropriate when occupancy is high and thus vector abundance and disease transmission are subsequently high. A recent agent-based entomological model suggested that fipronil treatment had greater potential to reduce sand flies when applied during periods when the vector population is high and thus more individuals are acquiring blood meals while fipronil efficacy is at its peak [40]. We observed 100% reduction in *Xenopsylla* spp. flea abundance when treatment was applied twice in early summer when the gerbil colony occupancy rate was ~77%. This level of efficacy was sustained until autumn. Given the rate of gerbil colony occupancy can differ in spring and autumn, it would be beneficial to perform another treatment in autumn and compare the efficacy with that of treatment performed in spring/summer, as [93] found that the effects of dusting prairie dog burrows on flea abundance were longer lasting in autumn when compared to spring. Taking into consideration the fact that socio-economic factors could limit the number of feasible program-initiated fipronil treatment applications, comparing treatments performed in spring and autumn could help to determine the optimal timing in which treatment should be applied. [94] suggested that satellite imagery can be used to estimate gerbil colony occupancy, with only 2% error when compared with direct observation, with the potential to predict plague outbreaks. If occupancy can be correlated with fipronil bait efficacy, it is then possible that satellite imagery, in conjunction with recent data sets regarding vector and host abundance, could also be used to determine the timing of treatment application.

As mentioned previously, no great gerbil or non-target wildlife fatalities were observed over the course of the experiment. Unlike other methods such as IRS or burrow dusting, the fipronil-based grain bait is applied orally to hosts, targeting blood feeding vectors exclusively, which reduces environmental exposure, minimizing contact with non-target wildlife or insects [52]. The gerbils within the treatment area appeared healthier than those within the control area (cleaner skin, fur), which could have possibly been a biproduct of bait application which reduced flea infestation from 100–0%. As previous researchers have already mentioned [55,69,95] we cannot be certain no non-target collateral occurred given observations were only performed above ground during the day. However, given the low fipronil concentration (0.005%) and an application rate of ~0.006 mg fipronil/m^2^, which was nearly 16x lower than what was used by [55], we can strongly suspect that negative non-target effects were reduced. The acute oral LD_50_ of fipronil in rodents is estimated to be 97 mg/kg body weight [46]. At this rate, a gerbil, weighing ~169–275 g [96], would need to consume ~>325–535 g bait in one feeding to attain the acute oral LD_50,_ a feat which would be improbable. Fipronil is more toxic to fish and at least three gamebird species (*Alectoris rufa*, *Phasianus colchicus*, *Colinus virginianus*) [97]. Exposure of fish to this treatment is highly improbable in the desert, and fipronil binds to soil and has low solubility in water suggesting reduced risk to aquatic organisms under field conditions [97]. The most vulnerable bird species to be studied is the northern bobwhite quail (*C*. *virginianus*), which is native to the United States, with an LD_50_ of 11.3 mg/kg. Bobwhite quail weighing on average between 140–170 g [98] would need to consume ~>30–40 g of bait in one feeding. This is less than what a mammal would have to eat, and less than what quail consume on average per day (~20 g) [99]. The most common bird species observed within the study plots, and the one with the closest association with the rodent burrows was *Oenanthe isabellina*, a small insectivorous passerine [100]. Fipronil has been shown to be less toxic to passerines such as *Spizella pusilla* (LD_50_: 1120 mg/kg) and *Taeniopygia guttata* (LD_50_: 310 mg/kg) [97]. It is encouraging that no bird mortalities were observed during the study, but also not surprising given the low concentration of fipronil in the bait (0.005%). To put it another way, while 100 kg bait was applied to the 771908 m^2^ plot during each application, the total amount of fipronil applied during each application was marginal (~5 g). In contrast, dusting with permethrin, the preferred control method in Kazakhstan, does not target the host explicitly and is effective if applied at rates of up to 2 g permethrin per individual burrow [41]. Previous largescale field trials conducted in Wyoming, during which fipronil was applied as a spray to 33% of two 347-ha treatment areas at a rate of ~0.4 mg/m^2^, determined the risk of fipronil to birds and non-target insect species to be far lower than that of alternative insecticidal compounds because it could be applied at rates of 100-200x less than alternative insecticides [101]. This is termed the “reduced exposure” approach, and strongly supports the argument that a 0.005% fipronil bait applied at a rate >65x lower than that of these Wyoming field trials should have reduced non-target effects. This does not however imply that non-target organisms should not be monitored, and more research should be conducted to determine the risk of this proposed treatment to various vertebrate species, and to investigate measures to further reduce non-target risk.

While efficacy against *Xenopsylla spp*. fleas exceeded requirements outlined by the EPA [73] caveats need to be addressed. Gerbils were collected 3x during Pre-Treatment (June 1-June 15), 1x during Treatment (June 16-June 21), 9x during Post-Treatment 1 (June 22-July 29), and 2x during Post-Treatment 2 (September 1–4). The trapping effort might be considered inconsistent and was largely a byproduct of trapping success and the availability of the individuals who were approved to perform the trapping. The flea numbers were also relatively low during the pre-treatment period. However, flea infestation was at 100% during pre-treatment and was reduced to 0% following treatment application. In contrast, flea infestation remained a constant 100% within the control plot throughout all study periods. While we are confident in our results, we proceed with caution, and recommend that future studies incorporate a more uniform trapping methodology and ensure that flea indices during the baseline period meet pre-determined criteria. We also note that while abundant fipronil laboratory and field data are available for other rodents such as roof rats [48], lesser-bandicoots [48], jirds [49], and black-tailed prairie dogs [55], laboratory-based fipronil studies involving great gerbils have not been conducted. While not a scope of this project, laboratory-based studies would be a useful addition to this field work to determine the fipronil half-life in the blood, feces and urine of great gerbils, as well as quantify its efficacy against both vectors. This information could aid in the design of future field studies and could be used to estimate parameters in predictive simulation modelling.

The benefits of oral fipronil treatment may not be limited to fleas and sand flies under the current conditions. It was observed during the experiments that tick genera (*Hyalomma*, *Rhipicephalus*, *Haemaphysalis*, and *Ornithodoros*) within the treatment area declined markedly during the experiment. Previous studies have indicated that fipronil is efficacious against *Ixodes scapularis* [53,54] which transmit *Borrelia burgdorferi*, the causative agent of Lyme disease. Future studies conducted in this region would benefit from evaluating the ability of fipronil-based treatment to reduce tick abundance, which, if determined to be efficacious, would increase the versatility of the bait, allowing for the potential to control several tick, flea and sand fly-borne diseases. The efficacy of fipronil bait against fleas infesting great gerbils in southeastern Kazkhstan, and black-tailed prairie dogs in northern Colorado [55], suggest that fipronil bait application could be effective in a number of unique biotopes. The great gerbil has a wide distribution in Asia, also being found in Afghanistan, Iran, Turkmenistan, Kyrgyzstan, Pakistan, Tajikistan, Uzbekistan, Mongolia, and China [102]. Environmental and geographic conditions within these countries inevitably differ from southeastern Kazakhstan. Therefore, we cannot say with absolute certainty that the results of this study would be applicable to all biotopes in Central Asia. However, we argue that these results indicate field trials in other plague and CL-endemic Central Asian countries are warranted.

## Conclusion

Our study suggests that fipronil-based bait may serve as a promising new tool for reducing disease vectors in southeastern Kazakhstan, and its potential should be further investigated. *X*. *gerbil minax* fleas were reduced by 100% after fipronil bait was applied twice (June 16, June 21), but efficacy against gravid female phlebotomine sand flies was inconsistent. In addition to the potential efficacy against fleas, our study suggests that low-concentration fipronil-based treatment may present reduced risk for both gerbils and non-target species in the area. While efficacy against fleas was significant, modifications in bait application timing and frequency will be needed to adequately reduce sand fly abundance. In conclusion, fipronil-based bait, applied in southeastern Kazakhstan, has the potential to reduce or potentially eliminate *Xenopsylla* spp. fleas if applied every 80-days, and could reduce gravid *Phlebotomus* spp. sand flies if applied more frequently. However, the study would benefit from a larger number of gerbils being sampled and more uniform trapping methodology. This form of treatment would likely be best applied in small areas where threats to humans or endangered species are of concern. Future studies should aim to monitor the vector populations over a longer period and should consider applying bait during more than one season, to determine the longevity of fipronil-based grain bait efficacy and determine the optimum timing and frequency of application. Additionally, future studies should consider the possibility of applying multiple treatments over multiple years to evaluate efficacy, insecticide resistance, and risk to non-target organisms. Fipronil-based bait may provide a means of controlling blood-feeding disease vectors in Central Asia and other regions globally.

## Supporting information

S1 Table Days of flea collection, sand fly collection, and fipronil application performed during all study periods. (XLSX)

S1 Video Great gerbils consuming fipronil bait during palatability test. (MP4)

S2 Table Environmental data reported May 1-September 30, 2016 in Bakanas Kazakhstan [60]. (XLSX)

S3 Table All fleas collected from gerbils and gerbil burrows within the treatment and control plot between June 1-September 4, 2016. (XLSX)

S4 Table Total *Phlebotomus* spp. and *Sergentomyia* spp. sand flies collected in CDC light traps June 1-July 29, 2016. (DOCX)

S5 Table Total non-gravid and gravid female *Phlebotomus mongolensis* collected at each trap sight per trap-night. Date of collection, study period, and collection number are all included.
(XLSX)
