# Supplementary material for: Field evaluation of a 0.005% fipronil bait, orally administered to Rhombomys opimus, for control of fleas (Siphonaptera: Pulicidae) and phlebotomine sand flies (Diptera: Psychodidae) in the Central Asian Republic of Kazakhstan
Source: PLoS Negl Trop Dis. 2018 Jul 25;12(7):e0006630. doi: 10.1371/journal.pntd.0006630 (PMC6059381; doi:10.1371/journal.pntd.0006630)
Supplement: S4 Table — (DOCX) [file pntd.0006630.s005.docx]

| **Plot ID** | **Total Sand Flies** | **Total Trap-nights** | ***Phlebotomus* spp.** | | | | ***Sergentomyia* spp.** | | | |
| --- | --- | --- | --- | --- | --- | --- | --- | --- | --- | --- |
|  |  |  | **Total** | Male | Non-gravid Female | Gravid Female | **Total** | Male | Non-gravid Female | Gravid Female |
| Treatment | 4031 | 270 | 3965 | 1757 | 1881 | 327 | 66 | 31 | 34 | 1 |
| Control | 5524 | 270 | 5378 | 2386 | 2401 | 591 | 146 | 48 | 97 | 1 |
| Total | 9555 | 540 | 9343 | 4143 | 4282 | 918 | 212 | 79 | 131 | 2 |
